# Diagnostic evolution of vestibular neuritis after long-term monitoring

**DOI:** 10.1016/j.bjorl.2021.02.004

**Published:** 2021-03-05

**Authors:** Sunghoon Kim, Yoon Kun Jung, Min Ji Kim, Kyu-Sung Kim, Hyun Ji Kim

**Affiliations:** Inha University College of Medicine, Department of Otorhinolaryngology-Head and Neck Surgery, Incheon, South Korea

**Keywords:** Vestibular neuritis, Caloric tests, Canal paresis, Final diagnosis

## Abstract

**Introduction:**

The diagnosis of vestibular neuritis is based on clinical and laboratory findings after exclusion of other disease. There are occasional discrepancies between clinical impressions and laboratory results. It could be the first vertigo episode caused by other recurrent vestibular disease, other than vestibular neuritis.

**Objective:**

This study aimed to analyze the clinical features and identify the diagnostic evolution of patients with clinically suspected vestibular neuritis.

**Methods:**

A total of 201 patients clinically diagnosed with vestibular neuritis were included in this study. Clinical data on the symptoms and signs of vertigo along with the results of vestibular function test were analyzed retrospectively. Patients were categorized in terms of the results of caloric testing (CP - canal paresis) group; canal paresis ≥25%; (MCP -minimal canal paresis) group; canal paresis <25%). Clinical features were compared between the two groups and the final diagnosis was reviewed after long-term follow up of both groups.

**Results:**

Out of 201 patients, 57 showed minimal canal paresis (CP < 25%) and 144 showed definite canal paresis (CP ≥ 25%). A total of 48 patients (23.8%) experienced another vertigo episode and were re-diagnosed. Recurring vestibular symptoms were seen more frequently in patients with minimal canal paresis (*p* = 0.027). Repeated symptoms were observed on the same affected side more frequently in the CP group. The proportion of final diagnosis were not different between two groups.

**Conclusions:**

Patients with minimal CP are more likely to have recurrent vertigo than patients with definite CP. There was no significant difference in the distribution of the final diagnoses between two groups when the vertigo recurs.

## Introduction

Acute vestibular syndrome consists of severe vertigo, nausea, vomiting, and postural instability. It is caused by unilateral vestibular damage to the peripheral or central nervous system.^1^ VN is one of the most common peripheral causes of acute vestibular syndrome. The main signs of VN are spontaneous horizontal torsional nystagmus beating away from the side of the lesion, abnormal head impulse test results on the involved semicircular canals, unsteadiness, with a falling tendency toward the lesion side and ipsilesional canal paresis (CP). In particular, unilateral CP has been the diagnostic hallmark of VN.^2^, ^3^, ^4^ Since VN has distinct clinical characteristics with laboratory tests performed later only when the patients can endure them, the initial diagnosis is based on history and physical examination alone. When the initial diagnosis was made only with clinical symptoms, these vertigo episodes may have been the first attack of other vestibular disorder such as Meniere’s disease or migraine. VN is generally known as a non-recurring disease,^4^ but a previous study has reported that the recurrence rate of VN reached up to 16% on 7–8 years follow-up.^5^

In the present study, we retrospectively investigated the final diagnosis after long-term follow-up of patients with clinically suspected VN presenting both with minimal CP and definite CP. We also analyzed how often these patients experienced relapsed vertigo or newly developed cochlear symptoms. We tried to discover the clinical features and diagnostic evolution of patients with clinically suspected VN.

## Subjects and methods

### Study population

Patients with acute spontaneous vertigo clinically diagnosed as VN and treated in our hospital between February 2008 and December 2015 were included in this study. The inclusion criteria for VN were: (1) complaint of a first attack of acute vertigo without cochlear symptoms (2) duration of dizziness lasting for over 1 h (3) spontaneous horizontal torsional nystagmus away from the lesion side (4) horizontal corrective saccades on the side of slow phase spontaneous nystagmus by head impulse test. The exclusion criteria were: (1) an experience of previous auditory symptoms such as hearing impairment, ear fullness, or tinnitus (2) history of migrainous symptoms related to recurrent vertigo (3) history of abnormal central nervous system findings. Patients with positional vertigo and abnormal findings on brain magnetic resonance imaging were also excluded.

### Audio-vestibular function tests

All patients were examined by experienced neurootologists. All patients underwent audiometric test and vestibular function test batteries including videonystagmography (VNG) tests, caloric test, rotation chair test and computerized dynamic posturography (EquiTest System, Neurocom Inc., Clackamas, OR, USA). Audiometric testing basically included a pure tone audiometry and speech audiometry using audiometer (GN Otometrics; Taastrup, Denmark). Videonystagmography tests, were performed to assess spontaneous, gaze evoked, positional, and positioning nystagmus. Oculomotor tests including saccades and optokinetic test were also performed.

A bithermal caloric test was performed. A bithermal caloric irrigator (3000 D; Life-Tech, Inc., Houston, TX, USA) that maintained a temperature of 30 °C for cold water, and 44 °C for the warm water (50 mL) for one minute each was used for the caloric tests. Each ear was irrigated with a constant flow alternating between hot and cold water. The maximum slow peak velocity of nystagmus was evaluated after irrigation. We used Jongkees’ formula to calculate CP, with a threshold for abnormal CP set at CP ≥ 25%. The patients were then divided into the minimal CP group (CP < 25%, n = 57) and the definite CP group (CP ≥ 25%, n = 144) based on the caloric test results.

### Follow-up

All patients were discharged after management of acute vertigo symptoms and instructed to re-visit if symptoms redeveloped. The medical charts were retrospectively reviewed. Patients were contacted by telephone by experienced otolaryngologists to discover further information. The patients were questioned regarding continuous or intermittent dizziness attacks after resolution of the first vertigo attack. The followup assessments consisted of clinical data collections including diagnostic re-evaluation. The features of vertigo symptoms such as type, duration, frequency, and associated symptoms were also assessed. We used a simple yes/no questionnaire to reduce the bias by expression of subjective symptoms. Otologic and neurologic examination and vestibular function tests were repeated in patients who revisited the clinic due to recurrent vertigo. Mean followup period was 36 ± 27 months. This study was approved by the International Review Board Committee (2020-04-022).

### Statistical analysis

All statistical analyses were performed with IBM SPSS ver. 18.0 (IBM Co., Armonk, NY, USA). We used the Pearson’s chi square test and Mann-Whitney U test for statistical analysis. A *p*-value of 0.05 was used as the value of statistical significance.

## Results

A total of 201 patients initially diagnosed and treated for VN in the emergency department at our institute were included. The patients consisted of 116 men and 85 women, aged 55 ± 10 years (range, 23–77 years). Among the patients, 57 showed minimal CP (CP < 25%) and 144 showed definite CP (CP ≥ 25%) in a caloric test. Patients were then categorized into two groups according to the results of caloric tests: MCP group (CP < 25%) and CP group (CP ≥ 25%).

The frequency of vertigo attack was recorded. In the MCP group, 17 patients (30%) complained of recurrent vertigo or newly developed cochlear symptoms after the first episode of vertigo fully subsided. They experienced an average of 2.1 ± 1.3 vertigo episodes up to five times. Each episode was included only if there was a symptom-free period. The second vertigo attack appeared on average of 31.2 ± 25.8 months after the first attack. Thirty-one patients (21.5%) from the CP group complained of recurrent vertigo or newly developed auditory symptoms. The average number of episodes was 1.3 ± 0.6, with maximum of three episodes in the CP group. Within 41.0 ± 28.9 months after receiving the first treatment for vertigo, a second attack appeared.

Patients in the MCP group experienced significantly more frequent vertigo symptoms (*p* = 0.027), but the time interval of recurring symptoms was not different between the two groups (Table 1).

**Table 1:** Analysis of the recurrent vertigo episodes.

	CP ≤ 25%	CP > 25%	*p*-Value
No. of episodes	2.1 ± 1.3	1.3 ± 0.6	0.027*
Duration (yrs.)^a^	2.56 ± 2.0	3.32 ± 2.3	0.386

CP, Canal paresis.

a Duration between first and second vertigo attacks.

* *p* < 0.05.

For the vestibular or cochlear symptoms in vertigo episodes, we identified the direction of the disease when the auditory symptoms such as tinnitus or hearing loss or nystagmus developed. Twenty patients showed recognizable directionality in recurrence: 4 patients from the MCP group and 16 patients from the CP group. Only one patient from the MCP group had recurrent symptoms on the same side of first vertigo attack. On the other hand, there were 14 patients (88%) who had repeated symptoms on the same side since the first vertigo developed. Repeated symptoms were observed on the same affected side more frequently in the CP group than in the MCP group (*p* = 0.032) (Table 2).

**Table 2:** Directionality of recurrent vertigo.

	CP ≤ 25%	CP > 25%	*p*-Value
Recurrent Direction			0.032*
Ipsilateral (%)	1 (25)	14 (88)	
Contralateral (%)	3 (75)	2 (12)	

CP, Canal Paresis.

* *p* < 0.05.

Five (29%) of the 17 patients who experienced recurrence of vertigo symptoms in the MCP group developed positional vertigo and were later diagnosed as benign paroxysmal positional vertigo (BPPV). Three patients (18%) revisited our clinic with recurrent episodic vertigo with auditory symptoms like tinnitus and ear fullness at the same time. They were later diagnosed with Ménière’s isease (MD). There were two patients with audiometrically-proven hearing loss. Six patients had complaints of headache during the vertigo attack but did not meet the ICHD-3 criteria for vestibular migraine. All of them were classified as probable vestibular migraine using the Barany classification.^6^ Three of them were diagnosed with recurrent VN.

Thirty-one patients from the CP group complained of recurrent vertigo or newly developed auditory symptoms. Out of the 31 patients, 8 (26%) were diagnosed with BPPV, 5 (16%) were diagnosed with MD, 12 (39%) were diagnosed with probable vestibular migraine, and 6 (19%) were diagnosed with recurrent VN. The proportion of followup diagnosis was not significantly different between the minimal and definite CP groups (Table 3).

**Table 3:** Proportion of final diagnosis of clinically suspected VN.

	CP ≤ 25%	CP > 25%	*p*-Value*
BPPV	5 (29)	8 (26)	0.522
MD	3 (18)	5 (16)	0.595
Probable vestibular migraine	6 (36)	12 (39)	0.534
VN	3 (18)	6 (19)	0.604

CP, Canal Paresis; BPPV, Benign Paroxysmal Positional Vertigo; MD, Ménière’s Disease; VN, Vestibular Neuritis.

* *p* < 0.05.

## Discussion

VN was diagnosed by excluding other diseases clinically, since there is no conclusive diagnostic test for VN. The diagnostic criteria of VN usually include the following conditions: sudden onset of vertigo without any other auditory symptoms, vertigo persisting for several hours to days, other neurologic symptoms excluded, and canal paresis on caloric testing.^3^ In this study, twenty eight percent of patients who had visited the emergency department were clinically diagnosed with VN and had normal a caloric response (CP < 25%) when the test was performed later during admission. These patients cannot be diagnosed as VN under current diagnostic criteria because even if clinical symptoms and signs such as nystagmus were consistent with VN, the caloric response was normal. This may be consistent with the findings of a previous study wherein a mild form of VN^7^ was reported. Another study also suggested that there may be an arbitrary diagnosis called mild vestibular deficit.^8^

In the present study, we investigated the long-term followup of the patients in the minimal canal paresis group if they developed other vestibular disease. We also determined if the patients showed different clinical characteristics from the definite VN patients who had profound canal paresis.

Molecular biological studies have presented evidence that VN can be caused by a latent reactivation of herpes simplex virus type 1 in the vestibular ganglia^9^ and that recurrent vertigo attacks are theoretically possible. There have been several conflicting studies on the recurrence of VN: some studies reported an overall recurrence rate of 1.9–16%.^5^, ^10^, ^11^ Recurrence of a typical VN is known to be uncommon, it is possible that the initial clinical presentation of recurred vertigo could be the first mono-symptomatic vertigo attack of another peripheral vestibular disease, such as MD or migraine, especially in the minimal canal paresis group patients. According to Bergenius and Perols, subjective symptoms of vestibular disturbance were present in up to 53% of VN patients 7–8 years after initial onset.^5^ Another previous study reported that the recurrence rate of vertigo of any cause in patients with VN was about 26%.^10^ In this study, 23.8% of patients (17 patients with MCP, 31 patients with CP) were identified as recurrent vertigo over the followup period. The recurrence rate was not different between the two groups.

There were reports about the appearance of BPPV in studies of recurrent vertigo after VN.^5^, ^10^, ^11^, ^12^ The prevalence of BPPV per study was reported to be 14–15.3%.^10^, ^11^ In our study, the prevalence of BPPV was five out of 57 (9%), and eight out of 144 (5%) in the MCP and CP groups, respectively. Bergenius and Perols found a higher value (31.6%) and they concluded that the high incidence of BPPV in VN patients was due to otoconia dislodged from the degenerated utricle due to VN.^5^ A similar finding suggests that patients treated with VN have some degree of vestibular damage, which has some effect on the instability of otoconia.^12^, ^13^ In this study, patients in the CP group had recurrent vertigo in the same side (same ear) which could be explained by the same hypothesis.

Ménière’s disease should be included in the differential diagnosis of recurrent vertigo. In the previous study, vertigo was the only initial symptom in approximately 25% of the patients with Meniere`s disease.^14^ The number of cases of MD in our study included both possible or definite MD, 3 (5.3%) in the MCP group and 5 (3.5%) in the CP group. In these patients, isolated vertigo had developed as a symptom of early-stage MD. We finally diagnosed MD in only 8 out of 201 (4%) patients. This result is inconsistent with the results of other previous studies, which found that 15 out of 45 patients (33%) had either possible or definite MD during the followup period (7–92 months).^8^ In this study, some patients were finally diagnosed with possible MD after several repeated isolated vertigo episodes, not the second.

Migraine can accompany true vertigo. Vestibular migraine is recognized as one of the most common causes of episodic vertigo. The clinical presentation of vestibular migraine is heterogeneous in terms of vestibular symptoms and duration of episodes.^15^ Nystagmus and caloric responses are known to be variable in patients with vestibular migraine. In this study, vestibular migraine accounted for the largest portion of recurrent vertigo. Eighteen patients (37.5%) showed vertigo with headache or migrainous symptoms. There was no difference in incidence between the two groups.

The proportion of final diagnosis was not different between the two groups. Patients in the MCP group also showed similar diagnostic evolution to those in the CP group in terms of vertigo recurrence. It could be considered that minimal canal paresis did not mimic other diseases but remained consistent with the existing study as mild type VN.

## Conclusion

Patients with minimal CP were more likely to have recurrent vertigo than patients with CP. Recurrent episodes of vertigo in patients with CP were predominantly on the same side as the previous vertigo. The proportion of the final diagnoses is similar in both groups when the vertigo recurs.

## Conflicts of interest

The authors declare no conflicts of interest.
